# Expression of a large coding sequence: Gene therapy vectors for Ataxia Telangiectasia

**DOI:** 10.1038/s41598-023-46332-4

**Published:** 2023-11-08

**Authors:** Tanja Hirch, Nadine Brander, Franziska Schenk, Simon J. Pöllmann, Janine Reichenbach, Ralf Schubert, Ute Modlich

**Affiliations:** 1 Division of Veterinary Medicine, RG Gene Modification in Stem Cells, Paul-Ehrlich-Institute, Langen, Germany; 2 Department of Gene and Cell Therapy, Institute for Regenerative Medicine – IREM, University of Zurich, Schlieren, Switzerland; 3 Department for Children and Adolescents, University Hospital Frankfurt, Goethe University, Frankfurt, Germany; 4 Deptartment of Somatic Gene Therapy, University Children’s Hospital Zurich, Zurich, Switzerland; 5 Competence Center for Applied Biotechnology and Molecular Medicine (CABMM), University of Zurich, Zurich, Switzerland

**Keywords:** Gene therapy, Genetic vectors

## Abstract

Ataxia telangiectasia is a monogenetic disorder caused by mutations in the *ATM* gene. Its encoded protein kinase ATM plays a fundamental role in DNA repair of double strand breaks (DSBs). Impaired function of this kinase leads to a multisystemic disorder including immunodeficiency, progressive cerebellar degeneration, radiation sensitivity, dilated blood vessels, premature aging and a predisposition to cancer. Since allogenic hematopoietic stem cell (HSC) transplantation improved disease outcome, gene therapy based on autologous HSCs is an alternative promising concept. However, due to the large cDNA of ATM (9.2 kb), efficient packaging of retroviral particles and sufficient transduction of HSCs remains challenging.

We generated lentiviral, gammaretroviral and foamy viral vectors with a GFP.F2A*.*Atm fusion or a GFP transgene and systematically compared transduction efficiencies. Vector titers dropped with increasing transgene size, but despite their described limited packaging capacity, we were able to produce lentiviral and gammaretroviral particles. The reduction in titers could not be explained by impaired packaging of the viral genomes, but the main differences occurred after transduction. Finally, after transduction of *Atm*-deficient (ATM-KO) murine fibroblasts with the lentiviral vector expressing Atm, we could show the expression of ATM protein which phosphorylated its downstream substrates (pKap1 and p-p53).

## Introduction

Ataxia Telangiectasia (A-T, OMIM #208900) is a rare multi-organ, monogenetic, autosomal recessive disorder with an incidence of 1 in 40,000–100,000 life births^1^, caused by mutations in the ataxia telangiectasia mutated (*ATM*) gene^2^. *ATM* is located at 11q22.3^3^, with an open reading frame of 9,168 bp and a corresponding protein kinase ATM of 350 kDa^2,4^. ATM plays a major role in DNA damage response, coordinates DNA repair, cell cycle arrest, apoptosis and genome stability^5–8^. Upon induction of double strand breaks (DSB) by e.g. radiation or oxidative stress, ATM is activated through autophosphorylation, leading to the dissociation of the inactive dimer and the activation of its kinase activity. Following this, ATM phosphorylates multiple downstream substrates (more than 700 protein targets identified^9^). In A-T, the activation of ATM and the downstream signaling cascades are disrupted or impaired, leading to a great variety of symptoms^10–12^. Progressive cerebellar degeneration resulting in ataxia, telangiectasia (dilated blood vessels), immunodeficiencies (impaired B- and T-cell development) following recurrent sinopulmonary infections and lung damage, radiation sensitivity, endocrine abnormalities premature aging, and a predisposition to cancer are the most prominent clinical signs^3,13,14^. Life expectancy is severely compromised, mainly due to chronic pulmonary diseases and malignancies^15^. The first symptoms are normally recognized in young children (2–3 years) and most patients are wheelchair bound in their second decade of life^1^. A retrospective cohort study of 240 A-T patients in France showed a 20-year survival rate of 53.4%^3^. Until today, there is no curative therapy for A-T patients. Most therapy options are symptomatic and supportive as immunoglobulin replacement, antioxidant therapy or glucocorticoid administration to treat the neurological symptoms^8^. To overcome hematologic abnormalities, allogenic hematopoietic stem cell (HSC) transplantation is an option to treat A-T patients that has been recently successfully explored^8,16^. Preclinical mouse models give further evidence of beneficial effects of HSC transplantation^8,17^. Autologous HSC gene therapy, using retroviral vectors, may be an alternative attractive option to allogeneic HSC transplantations, as no matching donor is needed and the risk of graft versus host disease is avoided. For this, retroviral vectors can be used for ex vivo transduction of autologous hematopoietic stem and progenitor cells (HSPC) and their transplantation back into the patient. The stable integration into the genome of HSPC guarantees the lifelong genetic modification of all blood cells^18^.

Due to its large size, ATM gene transfer using retroviral vectors is challenging. Foamy viral vectors are derived from retroviruses of the spumaretrovirinae subfamily which have the largest retroviral genome. They are therefore the most promising retroviral platform to deliver large coding sequences and were shown to be able to transfer about 12 kb^19,20^. Foamy viral vectors show a broad host and cell-type tropism and can efficiently transduce slowly cycling cells since foamy viral genomes can persist in transduced cells until they undergo mitosis^19,21^. Foamy viral vectors have an integration preference close to transcription start sites^22,23^ but with lower frequency than gammaretroviral vectors, and they show less read through transcripts^24^ than other retroviral vectors, suggesting a probably safer vector profile. As a drawback for gene therapy applications, foamy viruses cannot naturally be pseudo-typed with others than its foamy viral envelopes because of the important foamy viral Gag-Env interaction during particle formation^25^. But, with specifically engineered Gag variants pseudo-typing of foamy viral vectors with for example the well-known VSV-G (Vesicular stomatitis virus G protein) envelope becomes possible. This however lowers titers of these vectors, as compared to foamy viral vectors with their own envelopes^26^, and therefore we did not chose to use this system in this work.

Vectors derived from gammaretroviruses were the first vectors to be successfully employed in hematopoietic stem cell gene therapy. However, the first generation of gammaretroviral vectors contained the viral enhancer/promoter sequences in their long terminal repeats (LTRs). In combination with the gammaretroviral preference to integrate in promoter regions of active genes and close to CpG islands^23,27,28^, the insertional activation of cellular genes, including proto-oncogenes, induced leukemias in some of the patients treated in early clinical trials^29–31^. By implementing the self-inactivating (SIN) configuration and the use of cellular rather than viral promoters, the safety of gammaretroviral vectors could majorly be improved^32,33^. Gammaretroviral vectors have a packaging capacity of up to 10 kb^34^, while HIV-derived lentiviral vectors are described to be able to incorporate sequences with 8–10 kb^35,36^. It was also shown that lentiviral vectors can transfer gene cassettes of up to 12 kb, but with a strong negative impact on viral titers with increasing transgene size^37^. In contrast to gammaretroviral vectors, lentiviral vectors can efficiently transduce non-dividing cells, making them suitable for the transduction of HSCs. Lentiviral vectors are known to preferentially integrate within active transcription units^28,38^ but due to the use of cellular promoters in the SIN lentiviral vectors, they have much less potential to induce insertional oncogenesis^28,38,39^ and are nowadays the most often used vectors in clinical trials for monogenetic hematopoietic diseases^40–42^. Lentiviral and gammaretroviral vectors can be pseudo-typed with heterologous envelopes to broaden cell tropism (e.g. VSV-G)^27,34,43^.

In our study, to develop a gene transfer tool for *ATM*, we compared the three retroviral vector platforms for their potential to efficiently transfer and express ATM.

## Results

### Foamy viral vectors for gene transfer of Atm

**Figure 1:**
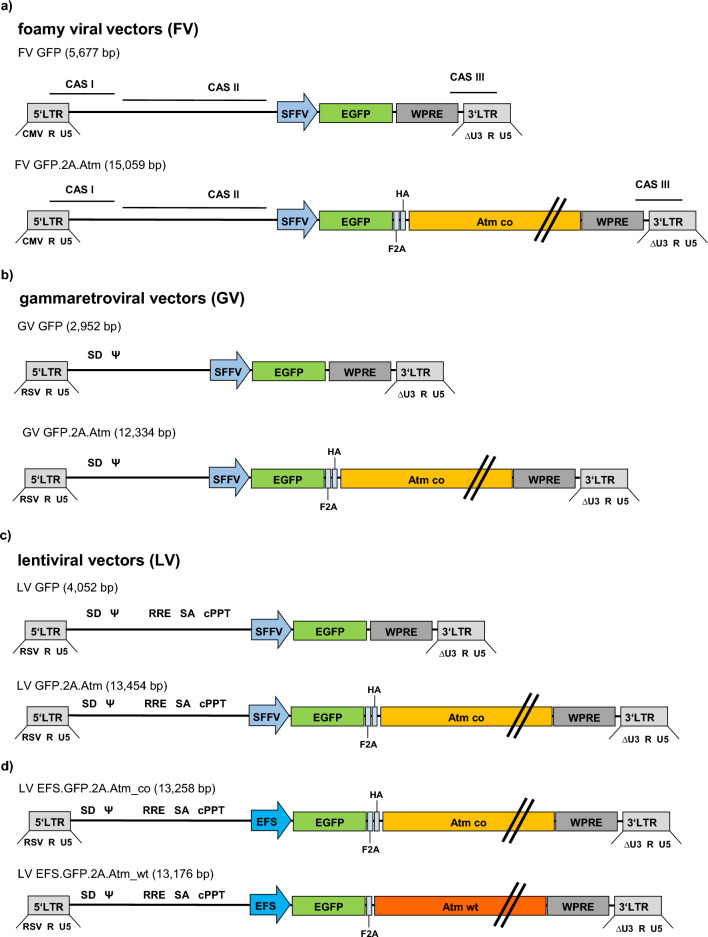
Schematic representation of each vector. (**a**–**c**) All small and large vectors were identical between the SFFV promoter and the WPRE element. (**d**) Lentiviral vectors with the EFS promoter and either a codon-optimized Atm sequence or the wild-type sequence; Respective sizes given from LTR to LTR. FV = foamy viral vector; LV = lentiviral vector; GV = gammaretroviral vector; LTR = long terminal repeats, ψ = packaging signal, RRE = rev responsive element, cPPT = central polypurine tract, WPRE = post-transcriptional regulatory element of the woodchuck hepatitis virus , PBS = primer binding site, SD = splice donor site, CAS = cis-acting sequences, RSV = Rous sarcoma virus promoter, CMV = cytomegalovirus promoter, SFFV = spleen focus forming virus promoter, EFS = elongation factor 1 alpha short promoter; F2A = 2A peptide from foot-and-mouth disease virus polyprotein, cleavage site, HA = hemagglutinin tag, co = codon-optimized; wt = wild-type sequence; Atm = murine Atm coding sequence.

We generated a self-inactivating foamy viral vector for Atm gene transfer by inserting a codon-optimized murine Atm cDNA into the foamy viral vector backbone^44,45^ under control of an internal spleen focus forming virus (SFFV) promoter. For detection of transgene by flow cytometry, GFP (green fluorescent protein) was co-expressed in the 5’ position, using a F2A protease cleavage site (consecutively named Atm vectors, Fig. 1a). As foamy viral vectors cannot be naturally pseudo-typed with VSV-G without gag-env modification to enable interaction, we first compared different foamy viral envelopes derived from the human prototype foamy virus or the macaque simian foamy virus using a GFP-coding control vector (Supplementary Fig. S1a). Furthermore, we optimized the transfection conditions to produce foamy viral vectors with high titers (Supplementary Fig. S1b).

**Figure 2:**
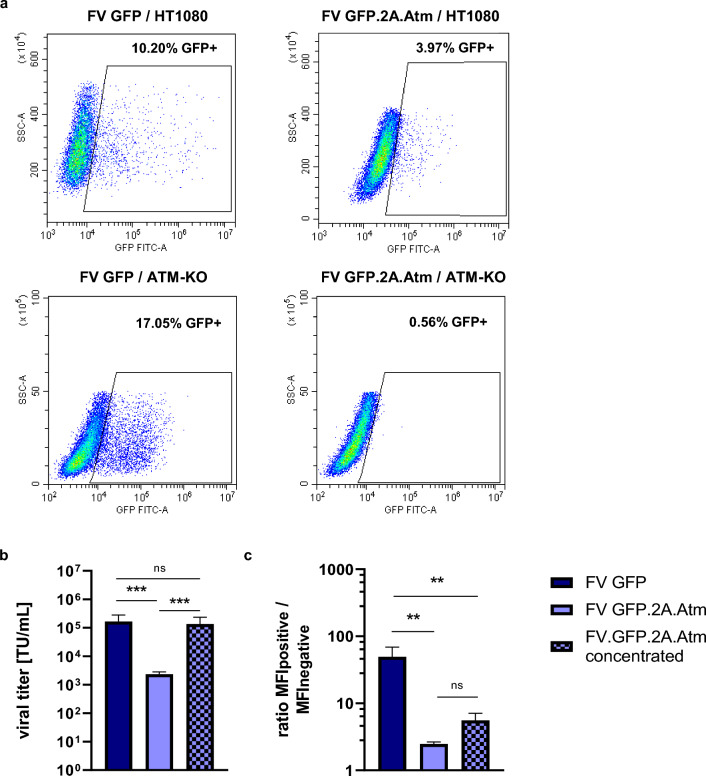
Viral titers and transduction efficiency of foamy viral GFP or foamy viral Atm vectors. (**a**) Representative flow cytometric plots of HT1080 cells or ATM-KO fibroblasts transduced with either FV GFP or FV Atm (concentrated) vectors three days post transduction. (**b**) Viral titers and (**c**) MFI in HT1080 cells transduced with foamy viral GFP vector, foamy viral Atm vector and concentrated foamy viral Atm vector determined three days post transduction; n = 3; for the calculation of titers of foamy viral Atm vector, only one value in each titration was used. TU/mL = transducing units / mL. All data represented as mean ± SD. (**b**) data were analyzed by one-way ANOVA with Tukey’s multiple comparison test; statistical analysis performed on log10 transformed data; (**c**) data were analyzed by one-way ANOVA with Tukey’s multiple comparison test **p ≤ 0.01; ***p ≤ 0.001; ns = not significant.

Using the best conditions and envelope, foamy viral GFP vectors were then produced with titers of 1.7 × 10^5^ TU/mL (transducing units / mL) determined by flow cytometry, while foamy viral Atm vectors achieved titers of only 2.5 × 10^3^ TU/mL (Fig. 2a + b). After two additional concentrating steps, foamy viral Atm vectors achieved titers of up to 1.6 × 10^5^ TU/mL (Fig. 2b). Expression levels (as measured by the mean fluorescence intensity, MFI) of GFP.2A.Atm were 17-fold lower compared to the GFP control vector (Fig. 2c). In addition, efficient transduction of target cells (*Atm*-deficient murine fibroblasts, ATM-KO) could not be achieved using foamy viral vectors (Fig. 2a).

### Lentiviral and gammaretroviral vectors for the expression of ATM

**Figure 3:**
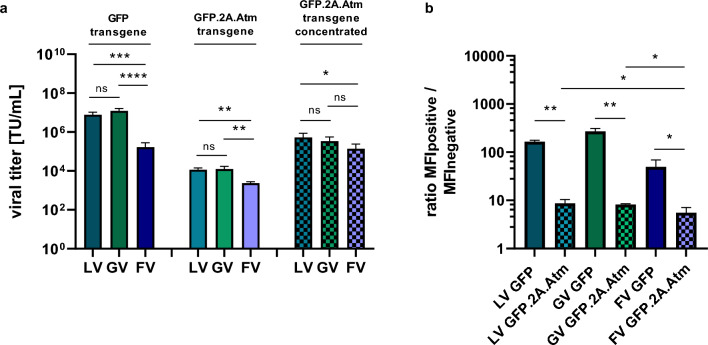
Viral titers of lentiviral, gammaretroviral and foamy viral Atm vectors are decreased compared to GFP control vectors. (**a**) Viral titers of lentiviral, gammaretroviral and foamy viral GFP control vectors as well as Atm vectors. For the GFP vectors, viral titers were determined with supernatants directly harvested and frozen after transient transfection; for Atm vectors, the viral titers were determined either with supernatants directly harvested and frozen after transient transfection or after two additional concentrating steps. (**b**) MFI values for the GFP-positive population divided by the MFI from the GFP-negative population; MFI determined three days post transduction of HT1080 cells; n = 3; TU/mL = transducing units / mL; MFI = mean fluorescence intensity. All data represented as mean ± SD. (**a**) data were analyzed by one-way ANOVA with Tukey’s multiple comparison test; statistical analysis performed on log10 transformed data; (**b**) data were analyzed by one-way ANOVA with Tukey’s multiple comparison test *p ≤ 0.05; **p ≤ 0.01; ***p ≤ 0.001 ****p ≤ 0.0001; ns = not significant.

As transduction of ATM-KO fibroblasts with the foamy viral Atm vector was not possible, we next asked whether Atm may be expressed from lentiviral or gammaretroviral vectors. Therefore, we generated lentiviral and gammaretroviral Atm vectors (Fig. 1b + c). As the packaging of lentiviral and gammaretroviral GFP vectors with the foamy viral envelope SEwt, that had shown the highest titers with the foamy viral GFP vector, was inefficient (Supplementary Fig. S2), we used VSV-G enveloped lentiviral and gammaretroviral vectors for our further studies. Lentiviral and gammaretroviral Atm vectors could only be produced with titers of 1.2 × 10^4^ TU/mL, compared to 7.7 × 10^6^ TU/mL and 1.2 × 10^7^ TU/mL for the smaller lentiviral and gammaretroviral GFP vectors, respectively. After concentrating steps, titers of 5.3 × 10^5^ TU/mL (lentiviral) and 2.5 × 10^5^ TU/mL (gammaretroviral) were obtained for the Atm vectors (Fig. 3a). The MFI in cells transduced with these Atm vectors was lower than the one achieved with the smaller GFP vectors. In addition, the lentiviral and gammaretroviral Atm vectors showed significantly higher expression intensities than the foamy viral Atm vector (Fig. 3b + Supplementary Fig. S3 + S4).

### Comparison of the three vector platforms

Since the gene transfer of the GFP.2A.Atm coding sequence with lentiviral and gammaretroviral vectors was unexpected due to their size, we systematically compared titers by flow cytometry and mean vector copy number (VCN) and the contribution of RNA containing particles in the vector preparations of the three vector platforms.

**Figure 4:**
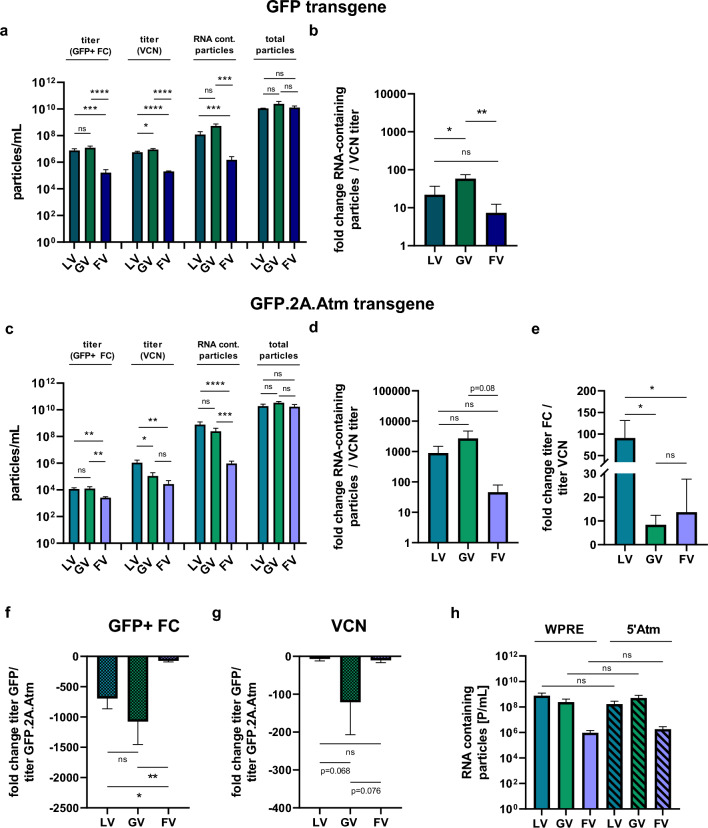
Comparison of viral titers determined by FC or VCN qPCR and RNA-containing particles. Viral titers determined by FC or VCN qPCR, RNA containing viral particles and total particle numbers of (**a**) lentiviral, gammaretroviral and foamy viral GFP vectors or (**c**) lentiviral, gammaretroviral and foamy viral Atm vectors. For FC analysis, HT1080 cells were analyzed for GFP positivity three days post transduction. For VCN qPCR, gDNA of transduced HT1080 cells were isolated seven days post transduction. (**b** + **d**) fold change of RNA containing particles to transducing particles determined with VCN qPCR for (**b**) GFP control vectors or (**d**) Atm vectors. (**e**) fold change in titer between FC and qPCR of Atm vectors. (**f**) fold change of viral titers between GFP control vectors and Atm vectors determined by flow cytometry, three days post transduction or (**g**) by VCN qPCR, seven days post transduction in HT1080 cells. (**h**) For the calculation of RNA-containing particles in the Atm vectors either primers binding on the very end of the integrated sequence (WPRE) were used or in the middle of the integrated sequence (5’Atm); n = 3. All data represented as mean ± SD. (**a**) and (**c**) data were analyzed by one-way ANOVA with Tukey’s multiple comparison test; statistical analysis performed on log10 transformed data; (**b**), (**d**)–(**h**) data were analyzed by one-way ANOVA with Tukey’s multiple comparison test *p ≤ 0.05; **p ≤ 0.01; ***p ≤ 0.001 ****p ≤ 0.0001; ns = not significant.

First, we compared small control vectors expressing only GFP. All three vector platforms produced 1–2 × 10^10^ particles/mL. The transducing titer measured by flow cytometry was 7.7 × 10^6^ TU/mL for the lentiviral vector and 1.2 × 10^7^ TU/mL for gammaretroviral vectors, but only 1.6 × 10^5^ TU/mL for the foamy viral vector (Fig. 4a). Similar titers were calculated when determining the mean VCN in transduced cells indicating that the integrated viral genome copies were actively expressing the transgene (Fig. 4a). We further measured the amount of RNA containing particles by quantitative PCR and detected significantly more in the supernatants of the lentiviral vector (22-fold), gammaretroviral vector (58-fold) and foamy viral vector (sevenfold) than were detected to be transducing (determined by VCN, Fig. 4a + b). Comparing the three vector platforms confirmed that the foamy viral vector was most successful in producing viral genome containing particles that were productively transducing target cells while the gammaretroviral vector was the least effective. However, the overall reached titers were lowest with the foamy viral vectors.

Titers of the Atm vectors determined by flow cytometry were markedly reduced compared to GFP vectors (700- to 1000-fold for lentiviral and gammaretroviral vector, 70-fold for foamy viral vector; Fig. 4c + f), however, integrated genome copies were readily detectable. Titers determined by the VCN were fivefold (lentiviral vector) and sevenfold (foamy viral vector) reduced, compared to the genome vector titer of the GFP control vectors, but 120-fold reduced for the gammaretroviral Atm vector (Fig. 4c + g). The amount of RNA containing particles on the other hand was much higher than were productively transducing, ~ 900-fold (lentiviral vector), ~ 2700-fold (gammaretroviral vector) and ~ 46-fold (foamy viral vector) higher (Fig. 4d). Because of the large coding sequence in the Atm vectors, we quantified in addition the amount of RNA in the particles based on an amplicon in the 5’ sequence of Atm, which will also detect truncated RNA genomes. However, we did not detect any differences between the amounts of RNA containing particles (Fig. 4h).

Taken together, the foamy viral vector showed the smallest ratio between RNA-containing particles and VCN titer and was therefore most efficient in integrating viral genomes of large size into target cells, while the gammaretroviral vector was the least effective. However, against our expectation, lentiviral and gammaretroviral vectors were able to transfer the transgene cassette of 10.2 kb, whereby the lentiviral Atm vector gave the best titers of all tested vectors and, therefore, appeared the most suitable for Atm gene transfer.

### Functional correction of ATM-deficiency by lentiviral Atm gene transfer

**Figure 5:**
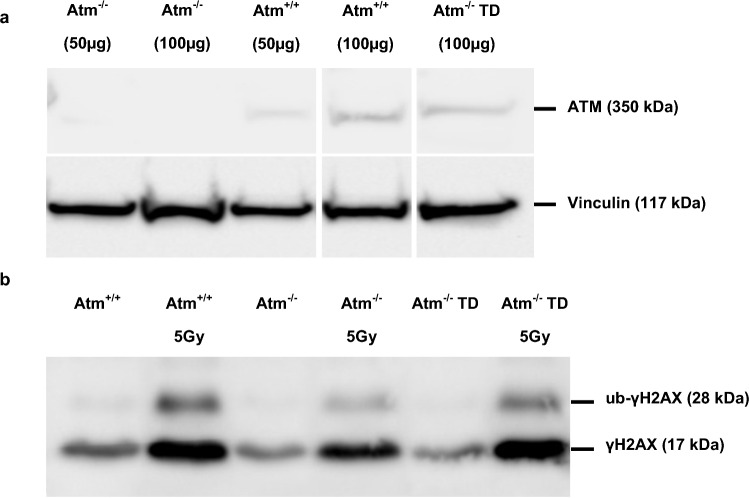
Reconstitution of ATM protein expression and restoration of phosphorylation in murine ATM-KO fibroblasts after transduction with lentiviral Atm vector. (**a**) ATM protein expression in murine fibroblast lysates (50 µg or 100 µg). Western Blot was probed with an anti-ATM antibody and an anti-Vinculin antibody as loading control. For detection of Vinculin, ATM blot was stripped and re-probed with the Vinculin antibody. (**b**) γH2AX/ ub-γH2AX Western blot of murine fibroblast lysates before and after irradiation. Western Blot was probed with an anti-γH2AX antibody. Atm^+/+^  = wt fibroblasts, Atm^-/-^ = ATM-KO fibroblasts, Atm^-/-^ TD = transduced ATM-KO fibroblasts with lentiviral Atm vector; ub-γH2AX = ubiquitinated γH2AX; Vinculin = loading control; Atm^-/-^ fibroblasts were isolated and immortalized from Atm-deficient mice; cropping of blot indicated by spaces between samples; full blots are shown in supplemental Fig. S6 + S7; n = 1.

We next investigated the potential restoration of ATM function in ATM-KO fibroblasts. Cells were transduced with lentiviral Atm vectors with a multiplicity of infection (MOI) of 0.5 (titrated on HT1080 cells). Three days post transduction, ATM-KO fibroblasts were 74% GFP-positive and ATM expression was 50% of wild-type levels, as detected by Western blot 24 days post transduction (Fig. 5a + Supplementary Fig. S5a).

The functionality of ATM was verified by testing the phosphorylation of H2AX, a histone involved in the responses to DSBs in wild type, ATM-KO and transduced ATM-KO fibroblasts 30 min after irradiation (5 Gy). Transduced cells showed a γH2AX level similar to wild type cells with an increased level of mono-ubiquitinated γH2AX compared to the ATM-KO control (Fig. 5b + Supplementary Fig. S5b + c). Mono-ubiquitinated γH2AX is a marker which is more related to DSBs than γH2AX, since γH2AX can also appear during apoptosis or severe chromatin damage^46^. We could thus show functional restoration of ATM protein in ATM-KO fibroblasts after transduction with lentiviral Atm vector.

### Clinically relevant lentiviral vector demonstrates correction of ATM deficiency

**Figure 6:**
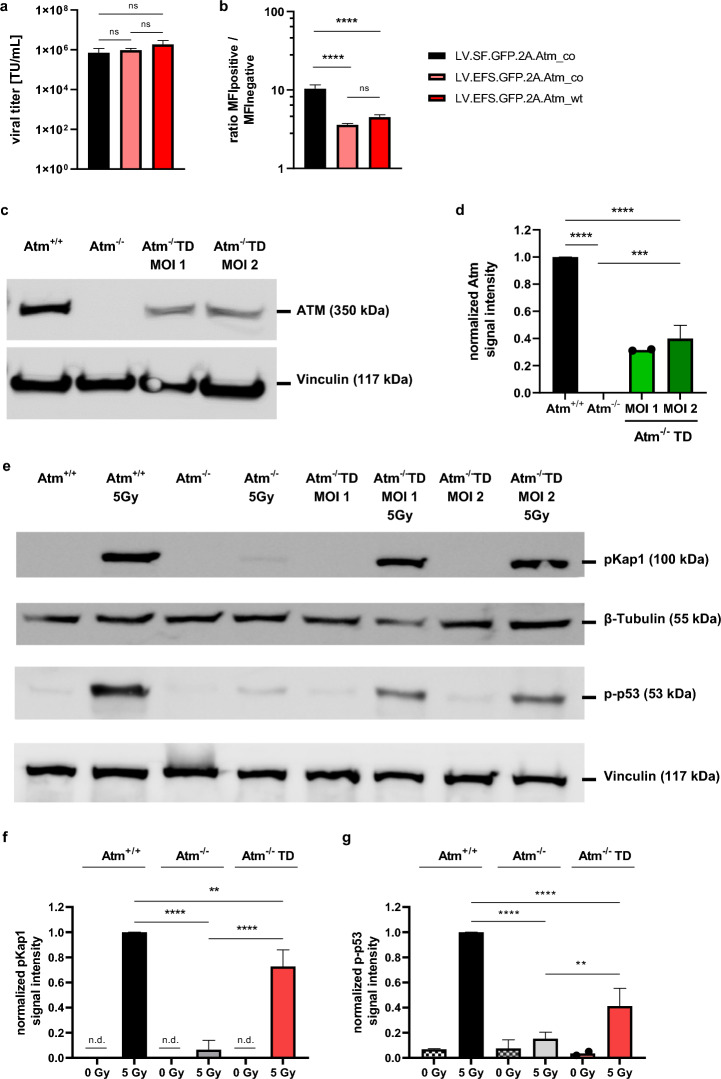
Reconstitution of ATM protein level and phosphorylation in ATM-KO cells transduced with LV.EFS.GFP.2A.Atm_wt vector. (**a**) Viral titers as well as (**b**) the ratio of the MFI; the MFI values for the GFP-positive population were divided by the MFI from the GFP-negative population; titers and MFI were determined three days post transduction of HT1080 cells; n = 3. (**c** + **d**) ATM protein expression in murine fibroblast lysates. (**c**) Representative Western Blot probed with an anti-ATM antibody and an anti-Vinculin antibody as loading control; (**d**) quantification of Atm signal intensity in murine fibroblast cell lysates; MOI 1: n = 2 (shown with individual data points); MOI 2: n = 3; (**e**) Representative p-Kap1 or p-p53 Western blot of murine fibroblast cell lysates. Western Blot was probed with either an anti-pKap1 antibody or an anti-p-p53 antibody, respectively. Vinculin and β-Tubulin were used as loading controls; for this representative WB, ATM-KO fibroblasts transduced with LV.EFS.GFP.2A.Atm_wt vector were 16.5% (MOI 1) and 19% (MOI 2) GFP-positive 10 days post transduction; protein lysates were produced on the same day; irradiated with 5 Gy where applicable. Quantification of (**f**) pKap1 and (**g**) p-p53 signal intensity in murine fibroblast lysates; n = 4. Atm^+/+^  = wt fibroblasts, Atm^-/-^ = ATM-KO fibroblasts, Atm^-/-^ TD = transduced ATM-KO fibroblasts with LV.EFS.GFP.2A.Atm_wt vector; full blots are shown in supplemental Fig. S8 + S9. All data represented as mean ± SD. a) and b) data were analyzed by one-way ANOVA with Tukey’s multiple comparison test **p ≤ 0.01, ***p ≤ 0.001, ****p ≤ 0.0001; ns = not significant; n.d. = not detectable.

For comparison of the vector platforms, vectors containing the SFFV promoter were used. Since this promoter is known to be able to induce malignant transformation by insertional activation of proto-oncogenes in the context of gene therapy^31,33,47^, we generated lentiviral vectors expressing the codon-optimized (co) Atm sequence (LV.EFS.GFP.2A.Atm_co), or the wild-type (wt) Atm sequence (LV.EFS.GFP.2A.Atm_wt) from the internal elongation factor 1 alpha short (EFS) promoter (Fig. 1d). All three vectors could be produced with similar titers, determined by flow cytometry (Fig. 6a). Furthermore, the lentiviral vectors containing the EFS promoter showed a significantly lower expression intensity of GFP compared to the vector expressing from the SFFV promoter (Fig. 6b).

Lastly, ATM-KO fibroblasts were transduced with the lentiviral vector containing the wt GFP.2A.Atm sequence driven by an internal EFS promoter. Transduced ATM-KO fibroblasts (15–20% GFP-positive) showed increased ATM protein levels compared to the ATM-KO control (35% of wt level, on day 10 post transduction, Fig. 6c, d) and an increased phosphorylation level of downstream substrates, namely p-Kap1 and p-p53 (Fig. 6e–g) after irradiation. These results demonstrated correction of ATM deficiency with a clinically relevant vector.

## Discussion

The aim of our study was to develop a retroviral gene transfer vector for the delivery of Atm. Because foamy viral vectors are known to be able to transfer large cDNAs, we initially chose this vector platform. Despite the previously reported superior ability of the foamy viral vector to transfer large virus genomes into target cells^19,20^, this platform was not suited due to low titers and inefficient transduction of ATM-KO fibroblasts. In contrast, the efficient gene transfer of the large GFP.2A.Atm coding sequence (10.2 kb) from the lentiviral and gammaretroviral vectors tested, was unexpected, especially in the light of previous reports that the packaging capacity of these vectors would be limited to 8-10 kb in case of lentiviral vectors and 10 kb in case of gammaretroviral vectors, respectively. This limit was described to be caused by either reduced incorporation of viral genomes into virions (RNA encapsidation)^37^ or by incomplete viral RNAs lacking the 3’ ends/LTRs, leading to incomplete reverse transcription^48^. In our hands, there was no detectable difference in the physical amounts of RNA-containing particles between the small GFP vectors and the Atm vectors, arguing against the idea of the presence of truncated viral genomes in viral particles. Furthermore, there was no difference in the amount of RNA-containing particles when comparing the calculation done after the qPCR amplifying a product in the 5’ Atm sequence or in the WPRE sequence. Our results are in line with the findings of Sweeney et al.^49^ demonstrating that the RNA content in lentiviral particles is not affected by transgene size.

The largest drop of titers for vectors with large transgene size was found when comparing RNA-containing particles and transducing particles. While in small vectors, 2–20% of RNA containing particles lead to productive integration (8.6% for lentiviral GFP vector, 2.4% for gammaretroviral GFP vector and 20.2% for foamy viral GFP vector), this was less than 0.32% for large vectors (0.002% for lentiviral Atm vector, 0.01% for gammaretroviral Atm vector and 0.32% for foamy viral Atm vector). Therefore, the main impact on functional vector titers happened after transduction in target cells although complete viral genomes appear to be packaged into particles. It is likely that either the step of reverse transcription could be hampered or the complete integration of the transgene into the genome.

In our hands, the best correlation between RNA containing particles and transducing titers was detected using the foamy viral vectors. However, the foamy virus has the unique characteristic to reverse transcribe it’s RNA during production of vector particles, i.e. in contrast to lentiviral and gammaretroviral vectors, reverse transcription is largely completed in virus producing cells and a prominent part of the functional genome in foamy viral particles is dsDNA^19,50^. It was shown that the contribution of viral genome DNA in foamy viral particles is between 6 and 20%^51^ and that this DNA is mainly contributing to productive transduction^52^. Translating this to our quantification, 15–50% of the foamy viral DNA genome containing particles led to integration in transduced cells. However, it has to be mentioned that we did not directly quantify the amount of DNA in foamy viral particles, our methodology only detected RNA in the particles.

When comparing titers determined by flow cytometry or by VCN qPCR, calculated titers were in the same range with both methods for the GFP transgene, independent of the vector system. For the Atm vectors, functional titers determined via flow cytometry were much lower compared to genomic titers determined by VCN qPCR. The lentiviral vector showed the largest difference between functional titer and the genomic titer (Fig. 4E) indicating that not all integrated vector genomes were active.

GFP expression levels of the Atm vectors (as determined by MFI) were significantly lower compared to the GFP vectors. This might be due to a) a longer time needed by a cell to translate a larger mRNA transcript (rate of translation elongation estimated with five amino acids per second^53^), meaning there may be simply less translated proteins coming from the GFP.2A.Atm transcripts; or b) a preferred translation of shorter mRNAs, as shown by Guo et al.^54^ in a computational model. In this study they concluded that translation initiation efficiency for short mRNAs is higher compared to longer transcripts which can be explained by economizing energy resources. The same is true for the transcription of genes, which is also an energy costly process. Castillo-Davis et al.^55^ showed that introns from highly expressed genes are significantly shorter that introns from genes expressed at a low level, suggesting that huge coding sequences introduced with retroviral vectors could be transcribed and translated with lower frequency compared to shorter genes. Lastly c), the stability of mRNAs and their half-lives in a cell can highly influence the expression of the related protein^56,57^.

Using lentiviral vectors containing the GFP.2A.Atm sequence, we could reconstitute ATM protein levels in ATM-KO fibroblasts (50% of wt level). Corrected cells showed an increase of phosphorylated H2AX after irradiation, demonstrating a functional reconstitution. For clinical translation, we designed lentiviral vectors containing an EFS promoter instead of the SFFV promoter and we demonstrated that ATM protein levels were restored to 35% of wt level and phosphorylation of downstream substrates (pKap1 and p-p53) was increased. Taken together, we developed a gene transfer tool for delivery of the large coding sequence of Atm, which proofed to be functional in restoring some aspects of ATM deficiency. Our study, therefore, paves the way for preclinical models to evaluate ex vivo HSC gene therapy for human patients suffering from A-T.

## Materials and methods

### Vector constructs

Gammaretroviral (SRS11.SF.GFP.pre)^58^ and lentiviral (pRRL.PPT.SF.GFP.pre)^59^ plasmids were kindly provided by A. Schambach, Hannover Medical School. The foamy viral vector (puc2MD9) was generated by D. Lindemann, TU Dresden^44,45^. The foamy viral transfer vector plasmid 2MD9.pre was generated by inserting the WPRE element (derived from the pSRS11.SF.GFP.pre vector) into the puc2MD9 vector. The WPRE element was inserted directly after the GFP transgene with the help of Gibson Assembly cloning. The transfer vector plasmids pRRL.PPT.SF.GFP.F2A.HA.Atm.pre, pSRS11.SF.eGFP.F2A.HA.Atm.pre and puc2MD9.SF.GFP.F2A.HA.Atm.pre were generated by standard cloning techniques. The vector plasmids contain the transgene sequence (GFP.F2A.HA.Atm, codon-optimized) between the *AgeI* and *NotI* or *BsrgGI* site. In addition, lentiviral vectors containing an internal EFS promoter, expressing either the codon-optimized sequence or the wild-type GFP.2A.Atm sequence were cloned. For the codon-optimized EFS vector, standard cloning techniques were used and the GFP.2A.Atm sequence were inserted into a lentiviral backbone containing an EFS promoter. For the cloning of the lentiviral Atm wild-type vector, Gibson Assembly was used to introduce the transgene sequence into the lentiviral backbone.

### Cell lines

The human kidney cell line HEK293T and the human fibrosarcoma cell line HT1080 were cultured in Dulbecco’s modified Eagle medium (DMEM, Sigma Aldrich), supplemented with 10% heat-inactivated fetal calf serum (FCS, Biochrom) and 1% L-glutamine (Life Technologies). The fibroblasts isolated from ear and tail tissue from Atm-deficient (129S6/SvEvTac-Atmtm1Awb/J) and Atm-competent mice^60^ were cultured in DMEM, supplemented with 10% heat-inactivated FCS (Biochrom), 1% L-glutamine (Life Technologies), 1% penicillin/streptomycin (AppliChem), 1% non-essential amino acids (NEAA, Gibco) as well as 50 µM mercaptoethanol (Life Technologies). All cells were cultured in a 5% CO_2_ humidified atmosphere at 37 °C. The isolation of fibroblasts from ear and tail tissue from mice has been described previously^61^.

### Generation of vector supernatants

5 × 10^6^ HEK293T cells were seeded in 10 cm plates, one day prior to transfection. On day of transfection, medium of cells was changed and 1% penicillin/streptomycin as well as 25 µM chloroquine (Sigma Aldrich) was added.

#### Gammaretroviral- and lentiviral vector production

Cells were transiently transfected at 60–70% confluency by calcium-phosphate transfection method. In case of lentiviral vector production, cells were transfected with either 10 µg (for lentiviral GFP vector) or 22 µg (for lentiviral Atm vector) lentiviral transfer vector plasmid, respectively, 10 µg gag/pol helper plasmid (pCDNA3.GP.4xC)^59,62^, 1.5 µg VSV-G helper plasmid (pMD2.G)^58,63^ and 5 µg Rev helper plasmid (pRSV_REV)^62^ per 10-cm plate. For gammaretroviral vector production, 10 µg (for gammaretroviral GFP vector) or 22 µg (for gammaretroviral Atm vector) gammaretroviral transfer vector plasmid, respectively, 15 µg gag/pol helper plasmid (pcDNA3.MLVg/p) and 1.5 µg VSV-G helper plasmid (pMD2.G)^58,63^ were added per 10-cm plate. Retroviral supernatants were collected 24 h and 48 h after transfection.

#### Foamy viral vector production

For foamy viral vector production, HEK293T cells seeded in 10 cm dishes were transiently transfected at 60–70% confluency by PEI (polyethylenimine) transfection method. Cells were transfected with 10.4 µg (for foamy viral GFP vector) or 22 µg (for foamy viral Atm vector) foamy viral transfer vector plasmid, respectively, 5.2 µg gag helper plasmid (pcoPG4)^45^, 2.4 µg pol helper plasmid (pcoPP wt)^64^ and 0.8 µg envelope helper plasmid (pcoSE wt)^65^. Foamy viral supernatants were collected 48 h and 72 h after transfection.

Supernatants were filtered through 0.45 µm sterile syringe filters to remove remaining cells and frozen at -80 °C, supplemented with 20 mM HEPES.

In some cases (when indicated), viral supernatants of the Atm vectors were concentrated in two additional steps. For this, ~ 220 mL viral supernatant was spun down at 50.000 × g for 2 h at 4 °C. Viral particles were resuspended in a volume of 0.6–0.8 mL. In a last step, the viral particles were transferred to an Amicon 0.5 mL 100 K concentrator column (Merck) and volume was reduced via centrifugation to a final volume of 100–150 µL leading to a final concentration step of ~ 1500-fold. Supernatants were aliquoted when needed and frozen at −80 °C.

Viral vector titers were determined by transducing HT1080 cells in the presence of protamine sulfate (4 µg/mL, Sigma Aldrich). For GFP vectors, 3.5 × 10^4^ HT1080 cells were plated into 12-well format and for Atm vectors 1 × 10^6^ HT1080 cells were plated in 10-cm plates 6–8 h in advance. Serial dilutions of the viral supernatant were incubated with the target cells. The percentage of GFP-expressing cells was determined by flow cytometric (FC) analysis 72 h after transduction with the BD Cytolex and analyzed with the CytExpert software. For the calculation of viral titers, samples with > 1% and < 30% GFP-positive cells were used. All transduction experiments were performed at least three times. Particle numbers in vector stocks were determined by nanoparticle tracking analysis (Nanosight NS300, Malvern Panalytical).

### Detection of the mean vector copy number (VCN) by PCR

VCN was determined on genomic DNA from HT1080 cells, which were isolated seven days post transduction using the NucleoSpin Tissue Kit (Macherey–Nagel, Düren, Germany). TaqMan real time PCRs were performed with the TaqMan Fast Advanced Master Mix (ThermoFisher), using specific primers and probes complementary to the WPRE element or the PTBP2 gene as housekeeping gene. VCN was calculated by normalization to the housekeeping gene and quantification by a plasmid standard. PCRs were run on the StepOnePlus Real-Time PCR System (Applied Biosystems, Waltham, MA, USA).

### Quantification of RNA containing retroviral particles

For quantification of RNA containing particles 500µL non-concentrated viral supernatant was resuspended in 500µL Trizol. RNA was isolated using the Direct-zol RNA MicroPrep Kit (Zymo Research, Irvine, CA, USA) following the manufactures’ protocol and afterwards cleaned up by RNA clean and concentrator Kit (Zymo research) to remove DNA contamination. Afterwards, the isolated RNA was reverse transcribed using RevertAid H Minus Reverse Transcriptase (Thermo Fisher Scientific) following the manufacturers’ protocols. Quantification of the WPRE element was performed as described above (VCN PCR) by using the primers and a probe complementary to the WPRE element. RNA containing particles were quantified by a plasmid standard. For quantification of RNA containing particles in GFP.2A.Atm vectors, primers and a probe binding to the 5’Atm were used in addition.

### Western blot

If applicable, cells were irradiated with a ^137^Cs-g ray source with 0.036 Gy/s (Gamma-irradiator OB29/4, STS GmbH, Braunschweig) and incubated for 30 min. Protein lysates from irradiated or non-irradiated cells were extracted using RIPA Lysis buffer (Abcam) together with a protease inhibitor cocktail (PIC, Carl Roth) and Phosphatase inhibitor cocktail (Roche) following sonication (sonicator: Bandelin GmbH).

In case of ATM, protein extracts (50 or 100 μg per lane, determined with Bradford Assay) were loaded onto a NuPAGE® Novex 3–8% Tris–Acetate Gel (Invitrogen), electrophoresed and electro-transferred onto a 0.45 µM PVDF membrane (Mini Trans-Blot® System, BioRad) following manufacturers’ recommendations for high molecular weight proteins. Membranes were blocked either with 5% non-fat milk in PBS/ 0.1% Tween20 or with 5% BSA in PBS/ 0.1% Tween20 for 1 h and incubated overnight with a primary antibody against ATM (1:1000, D2E2 Rabbit mAb, Cell Signaling, #2873). For detection of γH2AX, protein extracts (50 μg per lane, determined with Bradford Assay) were loaded onto a 12% Mini-PROTEAN® TGX™ Precast Gel (Bio-Rad), electrophoresed and electro-transferred onto a 0.2 µM PVDF membrane (Mini Trans-Blot® System, BioRad) following manufacturers’ recommendations. Membranes were blocked with 5% non-fat milk in PBS/ 0.1% Tween20 for 1 h and incubated overnight with a primary antibody against γH2AX (1:2500, pSer139 rabbit pAb, Novusbio, NB100-384). For the detection of pKap1 or p-p53, protein extracts (50 μg per lane, determined with a Bradford Assay) were loaded onto a 12% Mini-PROTEAN® TGX™ Precast Gel (Bio-Rad), electrophoresed and electro-transferred onto a 0.45 µM PVDF membrane (Mini Trans-Blot® System, BioRad) following manufacturers’ recommendations. Membranes were blocked with 5% BSA in PBS/ 0.1% Tween20 for 1 h and incubated overnight with a primary antibody against pKap1 (1:2500, Ser824; rabbit, monoclonal, BL-246-7B5, Bethyl Laboratries, A700-013) or p-p53 (1:2500, Ser15; rabbit, monoclonal, D4S1H, rodent specific, CellSignaling, #12,571). As loading controls, Vinculin (1:2000, W18245A, rat, monoclonal BioLegend, 938,402) or β-Tubulin (rabbit, polyclonal, CellSignaling, #2146) were used. Membranes were incubated with a secondary antibody (1:2000, HRP donkey anti-rabbit IgG (Polyclonal), BioLegend, 406,401, or 1:5000, HRP goat anti-rat IgG (Polyclonal), ThermoFisher, 31,470) for 1 h. After incubation with a substrate (Clarity Western ECL Substrate, Bio-Rad) chemiluminescence signal was recorded with an imaging system (ChemiDoc Imaging System, Bio-Rad) and analyzed with Fiji Software.

### Statistical analysis

Graphing and statistical analyses were done using GraphPad Prism version 9.5.0 (Graphpad software). Data are presented as mean ± SD, unless stated otherwise. For statistical testing between the means of 2 points, the 2-tailed, unpaired Student’s t test with Welch correction (*p < 0.05; **p ≤ 0.01; ***p ≤ 0.001; ****p ≤ 0.0001) was used. For comparison between two or more groups, we used 1-way analysis of variation (ANOVA) with Tukey’s comparison test (*p < 0.05; **p ≤ 0.01; ***p ≤ 0.001; ****p ≤ 0.0001); ns = not significant. Statistical details can be found in the main figure legends as well as in the supplemental figure legends.

### Supplementary Information

Supplementary Information 1.

Supplementary Information 2.

## Data Availability

The datasets generated during and/or analysed during the current study are available from the corresponding author on reasonable request.
