# Supplementary material for: Expression of a large coding sequence: Gene therapy vectors for Ataxia Telangiectasia
Source: Sci Rep. 2023 Nov 8;13:19386. doi: 10.1038/s41598-023-46332-4 (PMC10632516; doi:10.1038/s41598-023-46332-4)
Supplement: Supplementary file 1 — Supplementary Information 1. [file 41598_2023_46332_MOESM1_ESM.pdf]

abbreviation	description
A-T	Ataxia telangiectasia
ATM / Atm	ataxia telangiectasia mutated
cDNA	complementary DNA
co	codon-optimized
DSB	double strand break
dsDNA	double-stranded DNA
EFS	elongation factor 1 alpha short
F2A	2A peptide from foot-and-mouth disease virus polyprotein, cleavage site
FC	flow cytometry
FV	foamy viral
GFP	green fluorescent protein
GFP.F2A.Atm	GFP and Atm coding sequence fused via F2A site
GV	gammaretroviral
HSC	hematopoietic stem cell
HSPC	hematopoietic stem and progenitor cells
LTR	long terminal repeats
LV	lentiviral
ATM-KO	Atm-deficient
MFI	mean fluorescence intensity
MOI	multiplicity of infection
SFFV	spleen focus forming virus
SIN	self-inactivating
TD	transduced
TU/mL	transducing units / milliliter
VCN	vector copy number
VSV-G	vesicular stomatitis virus G protein
wt	wild-type
