# Supplementary material for: Expression of a large coding sequence: Gene therapy vectors for Ataxia Telangiectasia
Source: Sci Rep. 2023 Nov 8;13:19386. doi: 10.1038/s41598-023-46332-4 (PMC10632516; doi:10.1038/s41598-023-46332-4)
Supplement: Supplementary file 2 — Supplementary Information 2. [file 41598_2023_46332_MOESM2_ESM.pdf]

Expression of a large coding sequence: Gene therapy vectors for Ataxia Telangiectasia

Authors

Tanja Hirch, Nadine Brander, Franziska Schenk, Simon J. Pöllmann, Janine Reichenbach, Ralf Schubert, Ute Modlich*

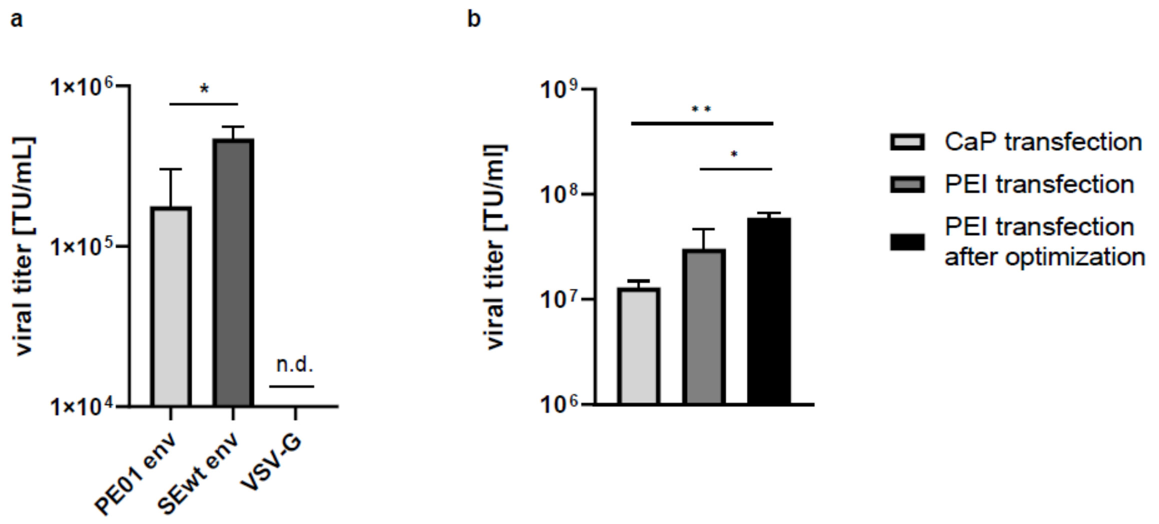

Supplemental Figure S1: preliminary optimizations to increase foamy viral vector titers

a) Transduction of HT1080 cells with foamy viral GFP vectors enveloped with either PE01 (prototypic foamy virus envelope), SEwt (simian foamy virus envelope) or VSV-G (vesicular stomatitis virus G protein); percentage of GFP+ cells was analyzed with flow cytometry three days post transduction; n = 3. b) Transduction of HT1080 cells with supernatants of foamy viral GFP vector produced with different transfections methods; viral supernatant was concentrated via ultracentrifugation; percentage of GFP+ cells was analyzed with flow cytometry three days post transduction. Calcium phosphate and PEI transfections were performed as described in the literature. For the optimization of PEI transfection; incubation time (10 min instead of 20 min), the amount of PEI reagent (60 µg instead of 46.6 µg) and the amount of plasmids (10.4:5.2:2.5:0.8 ratio) as well as time point for media change (no media change after 12 hours, first harvest of supernatant and addition of new media after 48 hours) were adjusted to increase viral titers; n = 3. CaP = calcium phosphate transfection; PEI = polyethylenimine transfection; n.d. = not detectable. All data represented as mean±SD. a) comparison of two means was analyzed by Student's t-test with Welch's correction *$p<0.05$. b) Data were analyzed by one-way ANOVA with Tukey's multiple comparison test *$p<0.05$, **$p\leq0.01$

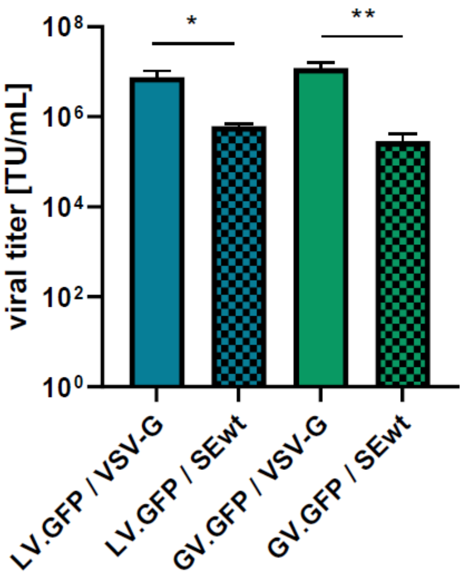

Supplemental Figure S2: decreased titers of lentiviral and gammaretroviral GFP vectors with foamy virus envelope SEwt

Transduction of HT1080 cells with lentiviral and gammaretroviral GFP vectors pseudotyped with either VSV-G (vesicular stomatitis virus G protein) or SEwt (simian foamy virus) envelope; percentage of GFP+ cells was analyzed with flow cytometry three days post transduction. All data represented as mean±SD. Data were analyzed by one-way ANOVA with Tukey's multiple comparison test *$p<0.05$, **$p \leq 0.01$

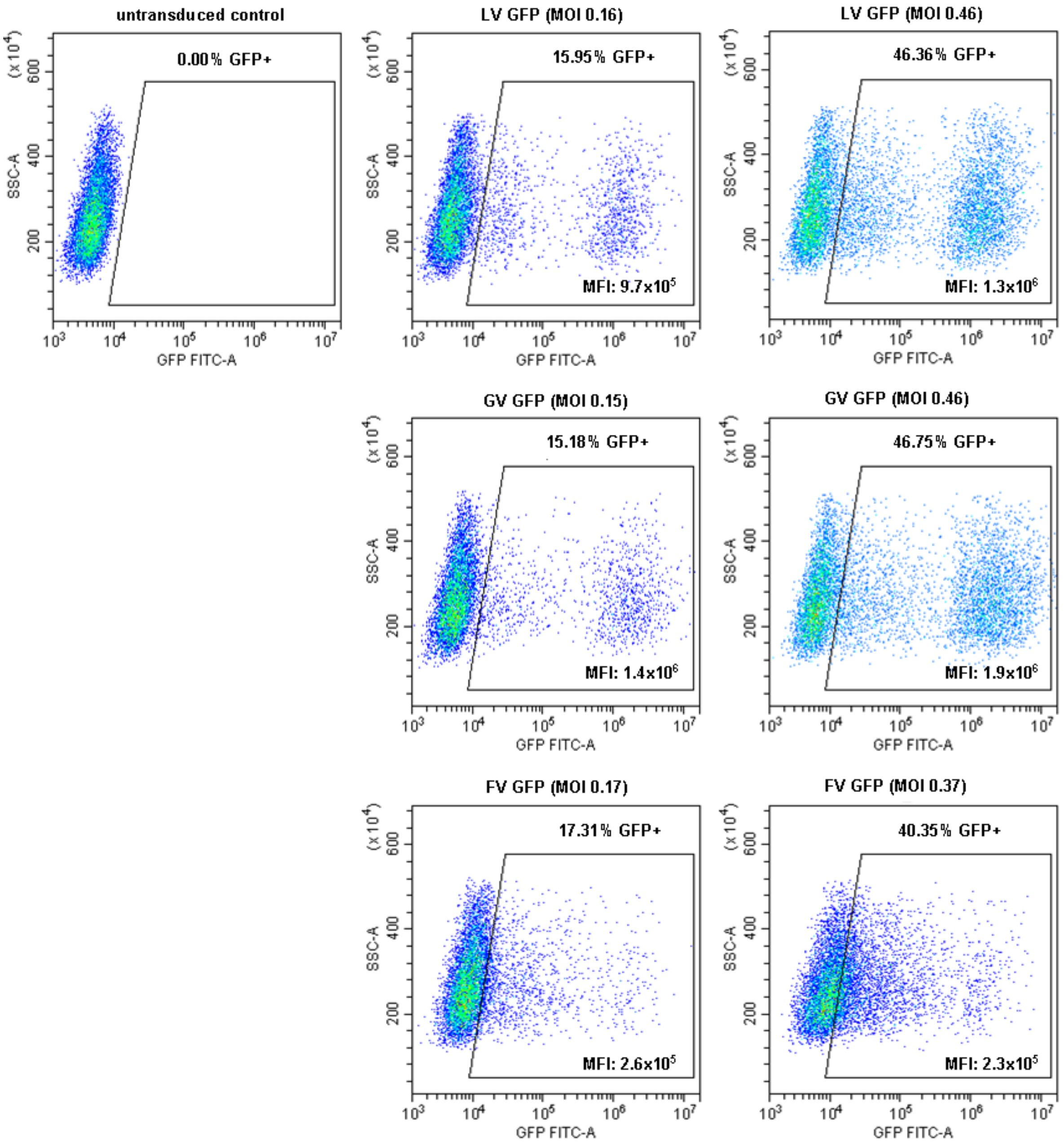

Supplemental Figure S3: representative flow cytometric plots of HT1080 cells transduced with non-concentrated lentiviral, gammaretroviral and foamy viral GFP vector supernatants

HT1080 cells were transduced with supernatants of non-concentrated lentiviral, gammaretroviral and foamy viral GFP vectors. The percentage of GFP+ cells as well as the mean fluorescence intensity of GFP was analyzed by flow cytometry three days post transduction; all transductions were analyzed on the same day.

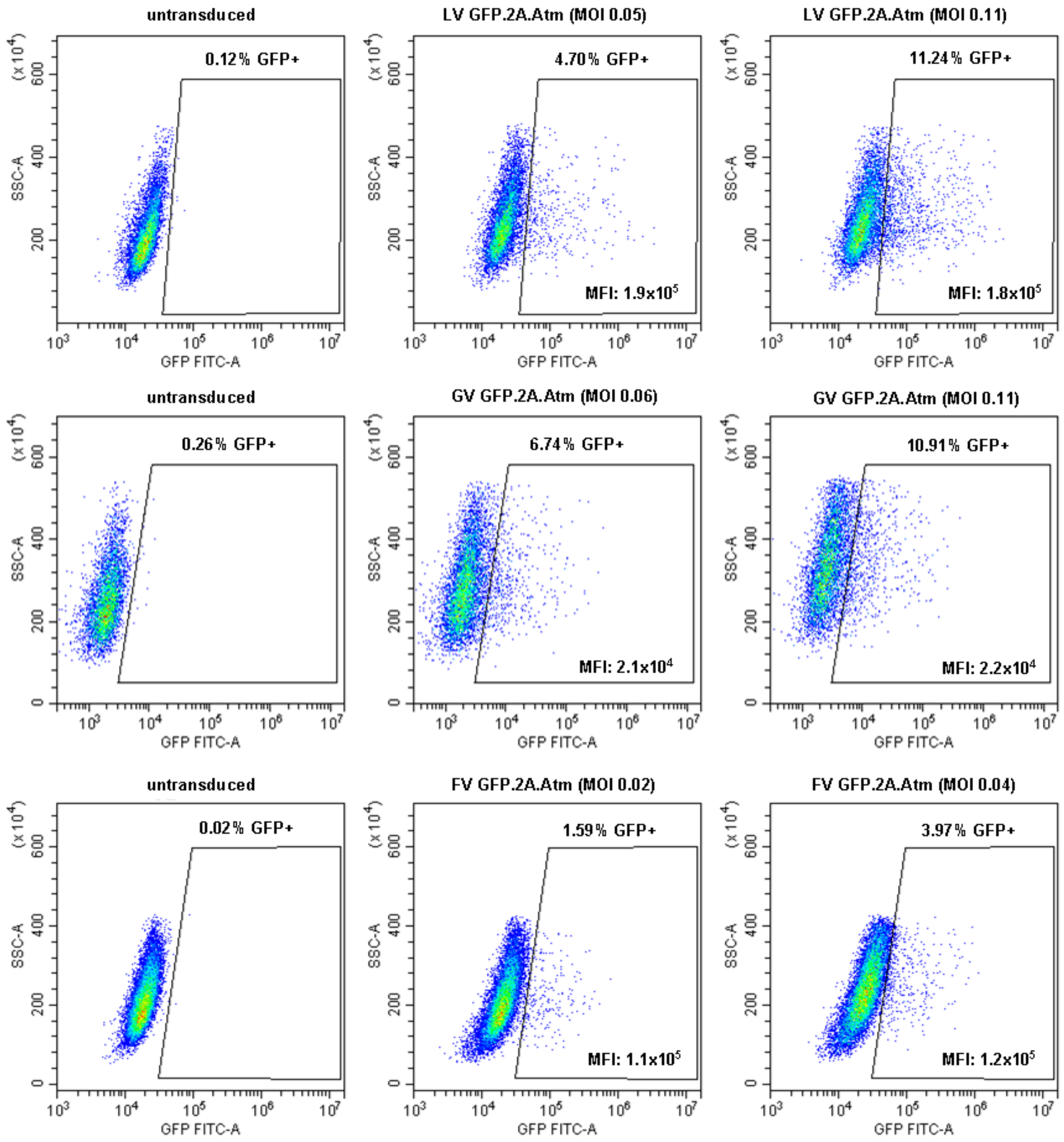

Supplemental Figure S4: representative flow cytometric plots of HT1080 cells transduced with concentrated lentiviral, gammaretroviral and foamy viral Atm vector supernatants

HT1080 cells were transduced with supernatants of concentrated lentiviral, gammaretroviral and foamy viral Atm vectors. HT1080 cells were transduced and analyzed on different days. Percentage of GFP+ cells as well as the mean fluorescence intensity of GFP was analyzed by flow cytometry three days post transduction.

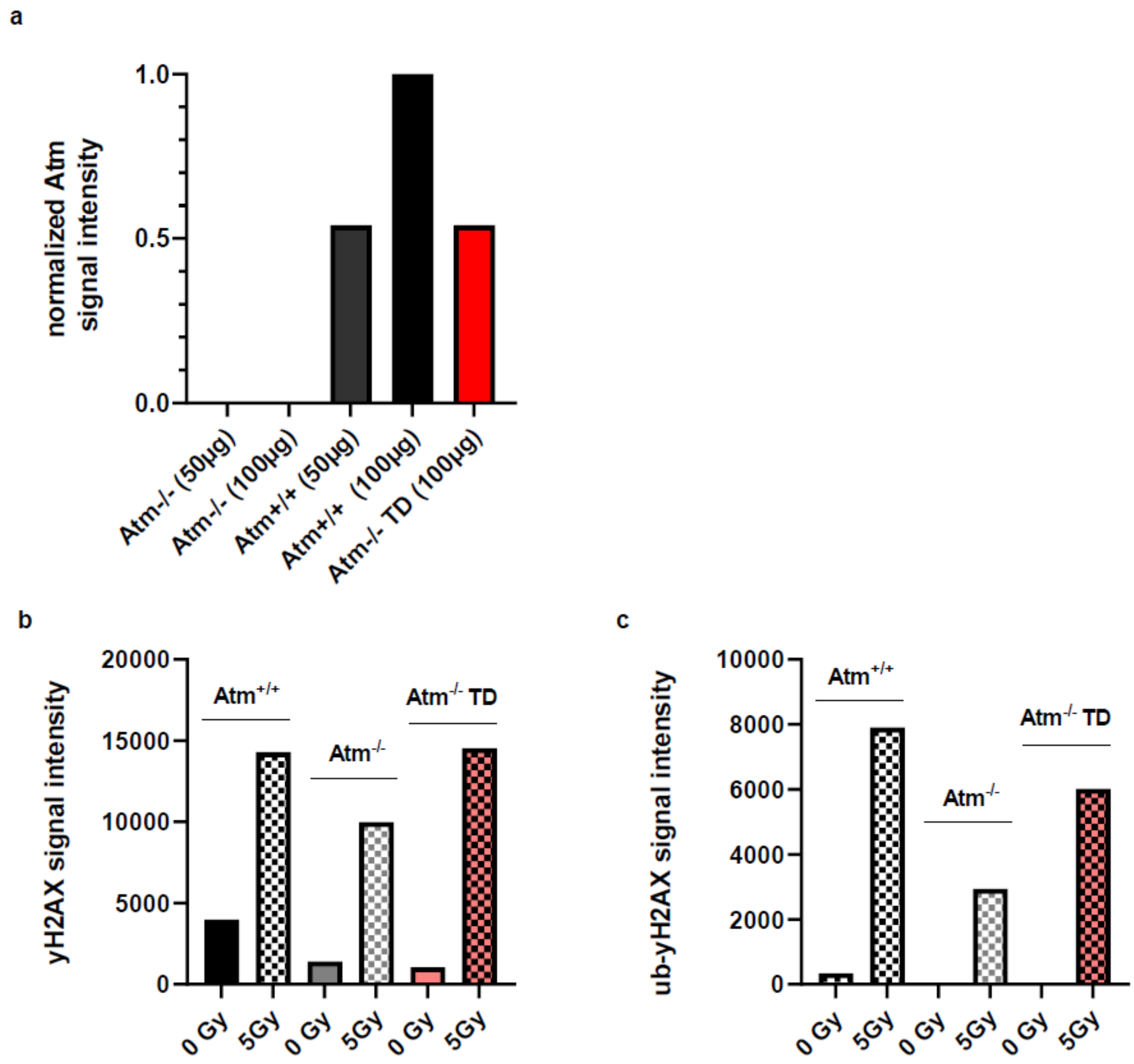

Supplemental Figure S5: quantification of Western Blot signals of fibroblast lysates transduced with lentiviral Atm vector

A) Quantification of ATM protein signal intensity in ATM-KO fibroblast lysates (of Western Blot shown in Figure 5a); Western Blot was probed with an anti-Atm antibody and an anti-Vinculin antibody as loading control. Quantification of B) γH2AX and C) ub-γH2AX signals in ATM-KO fibroblast lysates (of Western Blot shown in Figure 5b). Western Blot was probed with an anti-γH2AX antibody. ATM-KO fibroblasts transduced with lentiviral Atm vector with a MOI of 0.5 showed 74% GFP positive cells in flow cytometric analysis three days post transduction, lysates were produced 24 days post transduction; Cells were irradiated (when indicated) with 5 Gy. $Atm^{+/+}$ = wt fibroblasts, $Atm^{-/-}$ = ATM-KO fibroblasts, $Atm^{-/-}$ TD = ATM-KO fibroblasts transduced with lentiviral Atm vector; ub-γH2AX = ubiquitinated γH2AX; ATM-KO fibroblasts were isolated and immortalized from Atm-deficient mice; data represents n = 1.

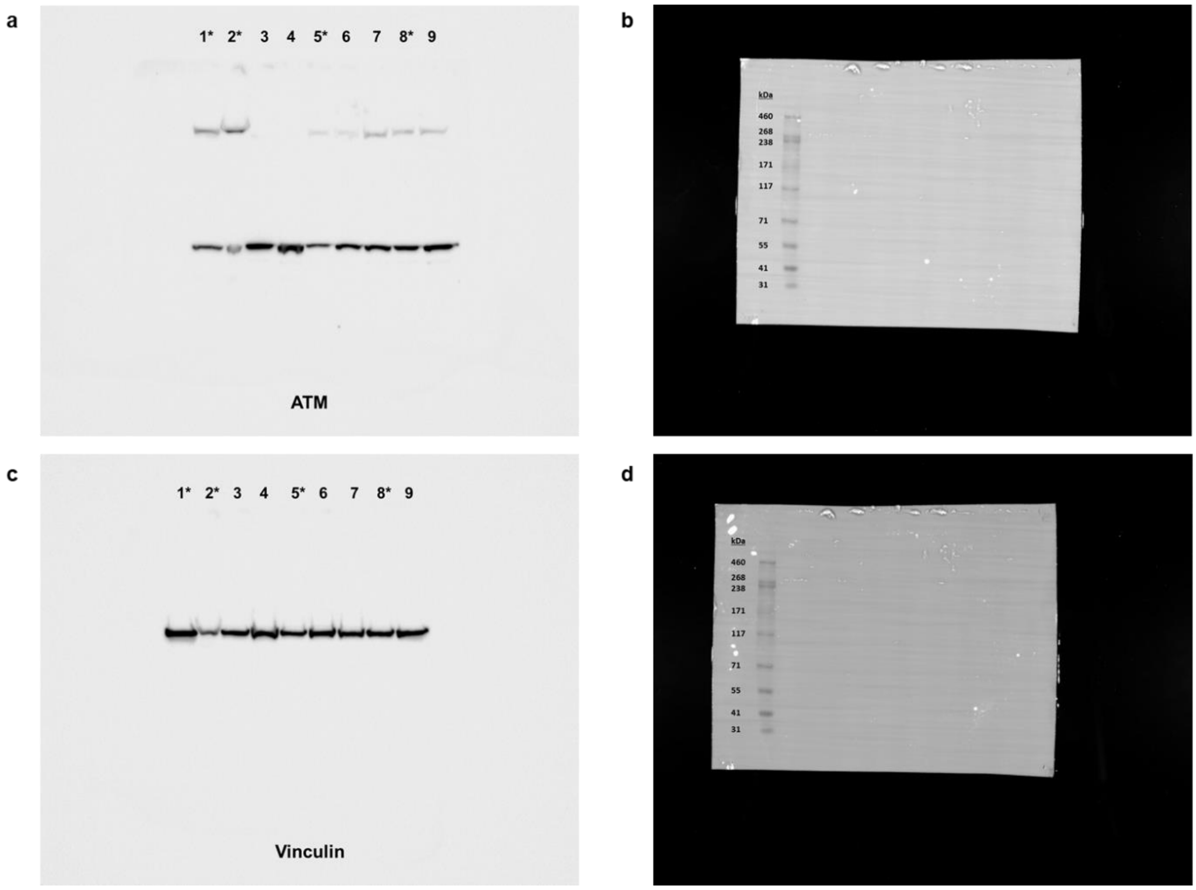

Supplemental Figure S6: full ATM Western Blot of Figure 5a

a) full blot of ATM immunoblotting; b) full blot of colorimetric ladder for ATM; c) full blot of Vinculin immunoblotting; d) full blot of colorimetric ladder for Vinculin; 1 = PC-3 cells (human prostate cancer cell line, as control); 2 = SC-1 cells (murine embryonic fibroblast cell line, as control); 3 = ATM-KO fibroblasts, 50µg loaded; 4 = ATM-KO fibroblasts, 100µg loaded; 5 = wt fibroblasts, 50µg loaded; frozen and thawed; 6 = wt fibroblasts, 50µg loaded; 7 = wt fibroblasts, 100µg loaded; 8 = ATM-KO fibroblasts transduced with lentiviral Atm vector MOI 0.1; 9 = ATM-KO fibroblasts transduced with lentiviral Atm vector MOI 0.5; *not shown in main figure

a

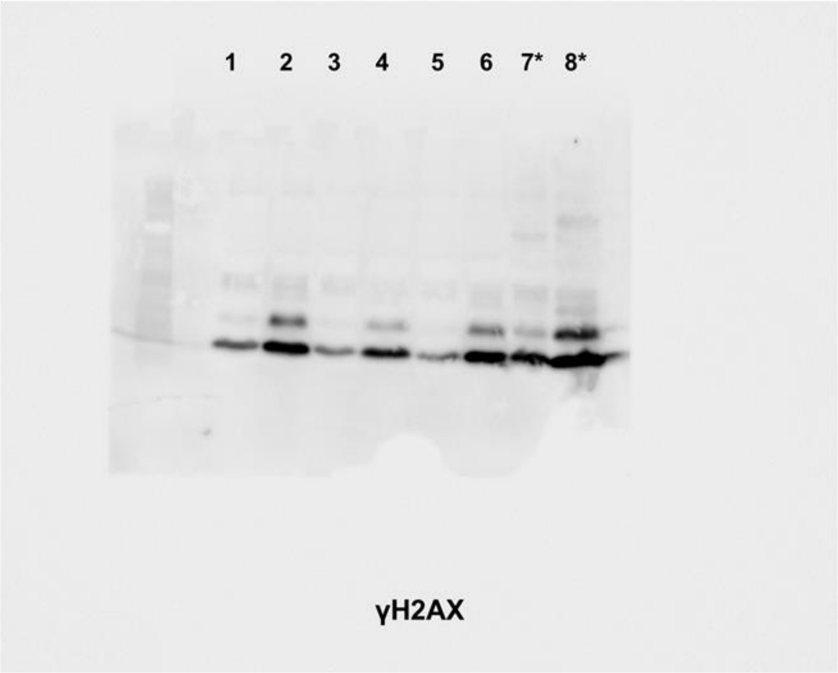

b

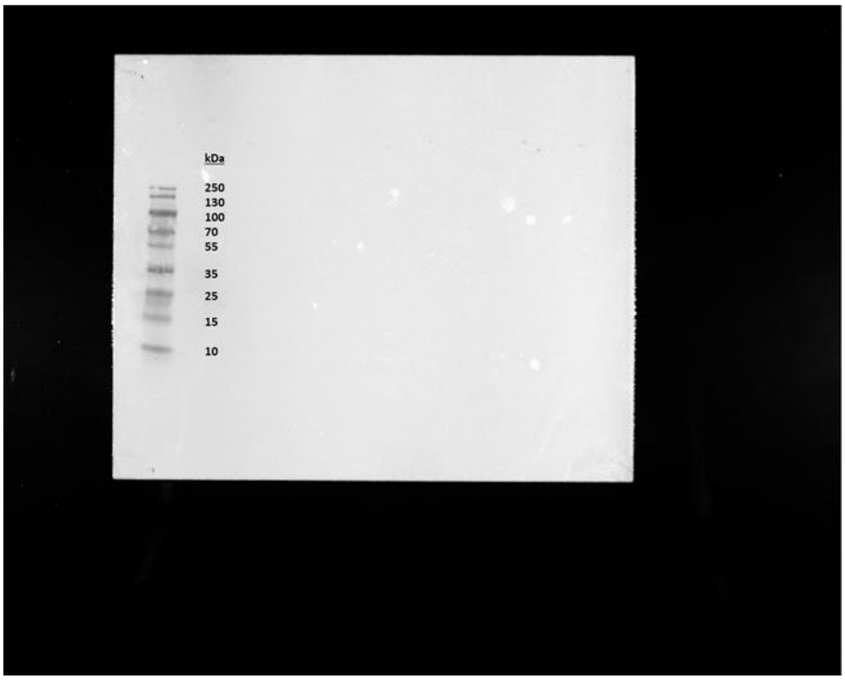

Supplemental Figure S7: full γH2AX Western Blot of Figure 5b

a) Full blot of γH2AX immunoblotting; b) full blot of colorimetric ladder; 1 = wt fibroblasts; 2 = wt fibroblasts after irradiation (5Gy); 3 = ATM-KO fibroblasts; 4 = ATM-KO fibroblasts after irradiation (5Gy); 5 = ATM-KO fibroblasts transduced with lentiviral Atm vector; 6 = ATM-KO fibroblasts transduced with lentiviral Atm vector after irradiation (5Gy); 7 = HT1080 (human fibrosarcoma cell line, as control); 8 = HT1080 (human fibrosarcoma cell line, as control) after irradiation (5Gy); *not shown in main figure

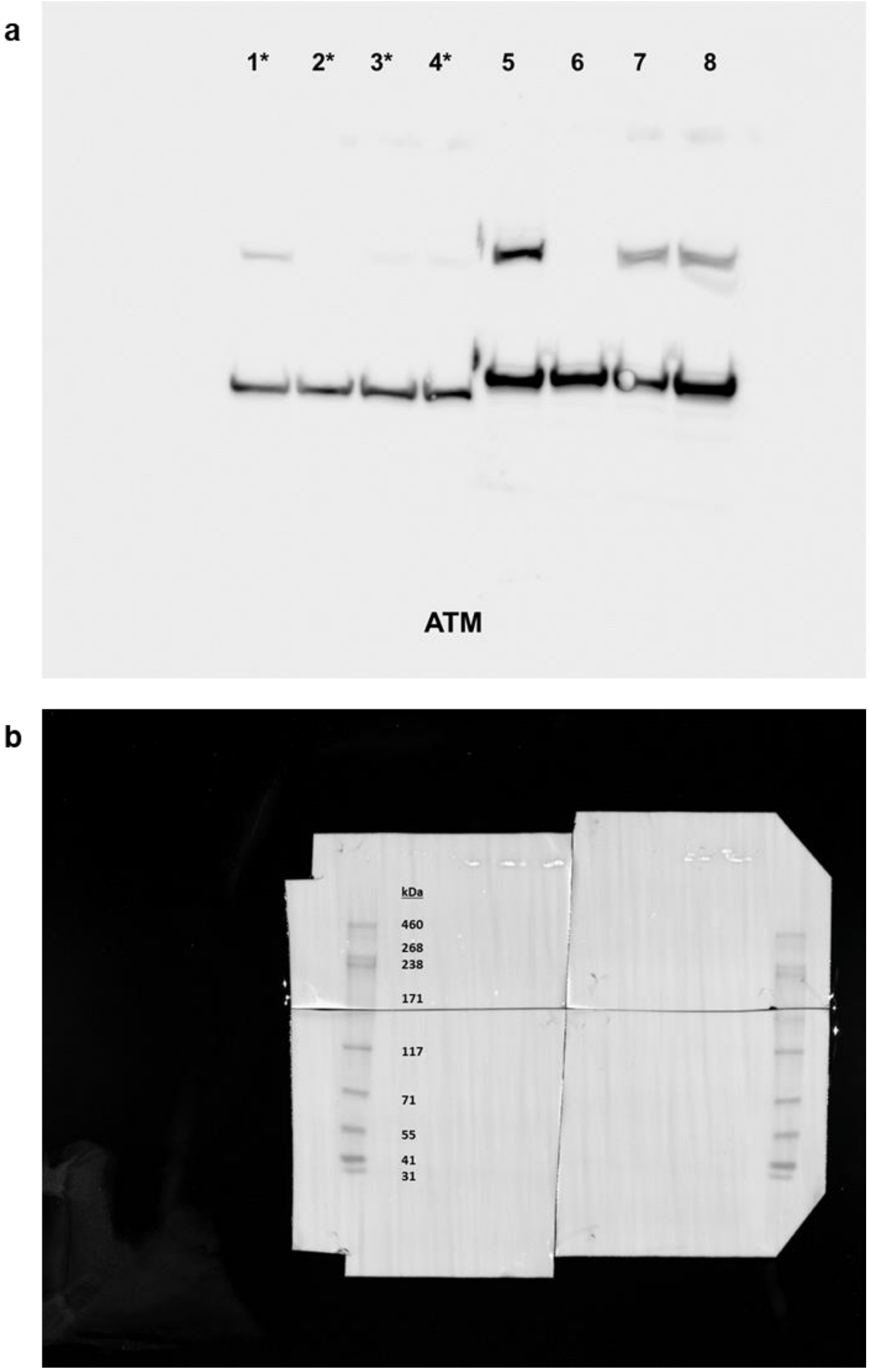

Supplemental Figure S8: full ATM Western Blot of Figure 6c

a) Full blot of Atm immunoblotting; b) full blot of colorimetric ladder; 1+5 = wt fibroblasts; 2+6 = ATM-KO fibroblasts; 3+7 = ATM-KO fibroblasts transduced with LV.EFS.GFP.2A.Atm_wt MOI 1; 4+8 = ATM-KO fibroblasts transduced with LV.EFS.GFP.2A.Atm_wt MOI 2; sample 1-4: membranes were blocked in TBST 5% milk; sample 5-8: membranes were blocked in TBST 5% BSA; for different blockings and incubations with different antibodies, the membrane was cut and re-assembled for imaging; *not shown in main figure

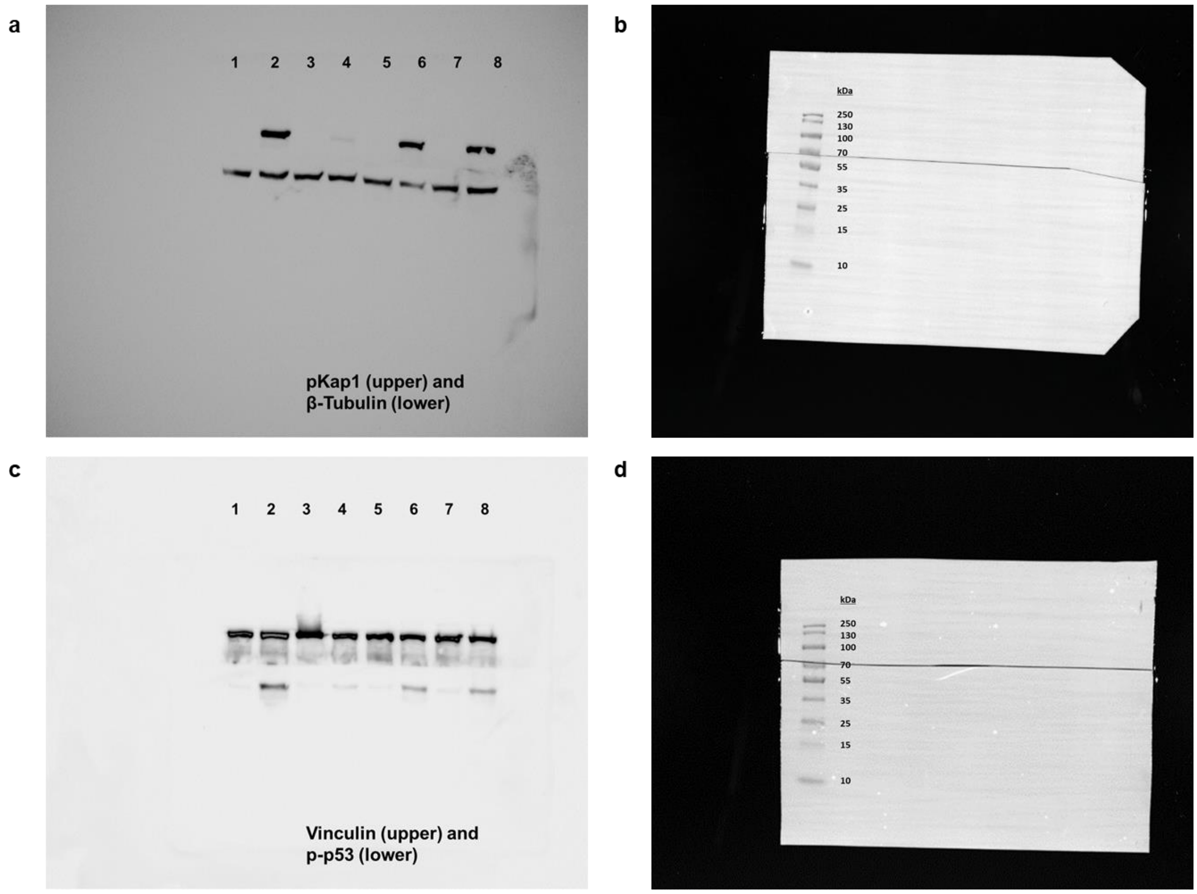

Supplemental Figure S9: full Western Blots of Figure 6e

a) Full blot of pKap1/ β-Tubulin immunoblotting; b) full blot of colorimetric ladder for pKap1/ β-Tubulin; c) full blot of Vinculin/ p-p53 immunoblotting; d) full blot of colorimetric ladder for Vinculin/ p-p53; 1 = wt fibroblasts; 2 = wt fibroblasts after irradiation (5Gy); 3 = ATM-KO fibroblasts; 4 = ATM-KO fibroblasts after irradiation (5Gy); 5 = ATM-KO fibroblasts transduced with LV.EFS.GFP.2A.Atm_wt MOI 1; 6 = ATM-KO fibroblasts transduced with LV.EFS.GFP.2A.Atm_wt MOI 1 after irradiation (5Gy); 7 = ATM-KO fibroblasts transduced with LV.EFS.GFP.2A.Atm_wt MOI 2; 8 = ATM-KO fibroblasts transduced with LV.EFS.GFP.2A.Atm_wt MOI 2 after irradiation (5Gy); for incubation with different antibodies, membranes were cut and re-assembled for imaging

Supplementary Table 1: sequences of primers and probes used for VCN qPCR or determination of RNA content in viral supernatants

**Target of amplification**	**Primer / Probe**	**5’-3’ Sequence**
WPRE	Forward	GAGGAGTTGTGGCCCGTTGT
	Reverse	TGACAGGTGGTGGCAATGCC
	Probe	5’-FAM-CTGTGTTTGCTGACGCAAC-3’-BHQ1
5’Atm	Forward	CTGGGGTGTTACTGCTACAT
	Reverse	ACTGCATCAGAGACTTAGCC
	Probe	5’-FAM- CATTATCACTGAAGACGAGG-3’-BHQ1
PTPB2	Forward	TCTCCATTCCCTATGTTCATGC
	Reverse	GTTCCCGCAGAATGGTGAGGTG
	Probe	5’-JOE-ATGTTCCTCGGACCAACTTG-3’-BHQ1

Supplementary Table 2: cycling parameters for qPCR

**Step**	**Temperature**	**Duration (s)**	**Cycles**
	50°C	120	1
Denaturation	95°C	20	1
Denaturation	95°C	5	40
Annealing	56°C	20	40
Extension	65°C	20	40
